# Inunction of Testosterone in Mental Disease

**Published:** 1942

**Authors:** A. Guirdham


### The Bristol Medico-Chirurgical Journal

*"Scire est nescire, nisi id me
Scire alius sciret."*

SPRING, 1942.

### INUNCTION OF TESTOSTERONE IN MENTAL DISEASE

BY

A. Guirdham, D.M., B.Sc., D.P.M.

This article is an account of three cases of mental disorder, two male and one female, treated by inunction with testosterone: in the two male cases with Perandren (Ciba), and in the latter case with Testoviron (Schering) ointment.

It is unfortunate that I have not full notes of the first case, which originally stimulated my interest in treatment of the psychoses by inunction with sex hormones. The other two I was able to observe in detail.

Case 1. The first patient was even more psychotic than the two described hereunder. He was undoubtedly a schizophrenic, alternating from extreme negativism to all attention, resistive stupor and bizarre catatonic postures to frenzies of catatonic excitement. He was in a deplorable physical condition owing to hypochondriacal delusions as to his gastric function. These delusions led him to abstain from all but minimal amounts of food, so that on admission it was considered that he might die of inanition if he did not eat more. He appeared to be constantly hallucinated, but could or would not describe his experiences.

Two inches Perandren (Ciba) ointment, containing two mgm. testosterone, was rubbed into his abdomen and in less than a week he put on four pounds, and was able to describe his hallucinations, and in little more than a week he was able, from a condition in which resistive stupor and catatonic gestures were main features, to describe important facts in the ætiology of his illness, such as a motor accident he had experienced. He was able to draw a diagram of this accident which had previously gone completely from memory.

The dosage of Perandren was increased to 4 inches (=4 mgm. testosterone). In about a fortnight he was active, dynamic and eager to discuss his condition, whereas formerly he had been like a vegetable, subject to gusts of irrational anger. Unfortunately he expressed a desire to go home. His relatives endeavoured to dissuade him, failed, and allowed him to have his way. I felt it inconceivable that such a sudden change in a schizoid state could be due to the specific treatment applied, and would have been inclined to regard it as an infrequent and unusually dramatic type of remission, except that I felt the remarkable increase in weight and appetite, and the patient's greatly improved appearance in so short a time, betokened some kind of massive constitutional change. I urged the relatives to continue with the treatment by the ointment, and the family doctor very carefully and competently supervised the patient. Less than a month after discharge he was eating, drinking and sleeping normally, and was swimming, walking and cycling. His mother described his improvement as really wonderful and a week later (*i.e.* less than five weeks after discharge) she described the patient as better than he had been for two years. At this juncture he found work as a market gardener, and three months later he was working regularly, cycling seven miles daily each way to his work, playing games and dancing, and was four stones heavier than on admission to hospital.

**Case 2.** The patient, a male, was aged 43 and was admitted in a state of profound melancholia. Psychotic symptoms predated admission by a year. He was profoundly retarded, he never spoke unless addressed and then in a whisper and at very great intervals. At times he remained mute. He had delusions that he had venereal infection (this possibility was completely excluded). His physical health was bad. He was emaciated and weighed no more than 6 stone 2 lbs.

A week after admission he was given methyl-testosterone, 5 mgm. daily, by mouth. This was continued for over three weeks. It produced, instead of his almost mute state, an agitated melancholic condition, in which he wandered round the room with a lost stare and proved extremely difficult about his food, eating minute quantities after great persuasion. The general tremor he showed on admission was accentuated, in addition to which he showed movements indicative of instability of the *corpus striatum*, even to the extent of a "pill-rolling" tremor on the right side.

Treatment was stopped for almost a month during which time the tremor and motor restlessness diminished, to reappear again on resumption of treatment with methyl-testosterone in the same dosage as before, but on alternate days. Again treatment had to be abandoned after just over a week because of tremor, excitability and motor restlessness. Sympathetic stimulation was noticeable in flushing of the skin of the face and in pupil dilation. The end result of oral treatment with methyl-testosterone was that after three months there was no improvement in the patient's condition. His weight was unaltered, he was in a particularly unsatisfactory state and seemed marked for chronic unrecoverable melancholia.

The patient was then treated by inunction with Perandren ointment. The skin of the abdomen was first cleansed with soap and water, dried carefully and the ointment rubbed in slowly, gently and thoroughly, the rubbing taking from ten to fifteen minutes, and being continued till the skin was no longer greasy. The rubbing was performed by a nurse wearing rubber gloves. The patient was given 2 inches of the ointment (=2 mgm. testosterone) daily. Though the patient's weight had remained unchanged after three months, he put on 2 lbs. at the end of a week's treatment. After a fortnight the amount of ointment used was increased to 4 mgm. testosterone daily. After five weeks the patient had gained 10 lbs. In the second week he began to take his food better, and at the end of a month he was extremely doubtful as to the truth of the abnormal ideas he had previously held so firmly. At this time (*i.e.* after four weeks' treatment) the amount of ointment was increased to 6 mgm. testosterone. At the end of six weeks his delusional ideas had completely gone. He was a moderately depressed and retarded but rational man. At the end of two months he had put on one stone in weight, was much more cheerful, and was beginning to occupy himself and able to smile.

In a little over three months his mood was completely changed. He was infinitely more cheerful, able to express optimism and the tempo of his thoughts changed definitely. His weight had increased by 1 stone 5 lbs. The dosage of ointment was increased to 8 mgm. testosterone daily. Previously inert, passive and accepting any orders given him, he became definitely dynamic and wished to go home, but was sufficiently reasonable to realize the advisability of staying till his improvement was more consolidated. In a further six weeks, when he left hospital, he had in all increased weight under treatment with Perandren by 1 stone 11 lbs., and was active, walking two hours a day without complaint, being confident, communicative, unretarded and without any trace of delusions. The improvement recorded occurred in the space of four and a half months' treatment with Perandren.

The patient has kept in touch with me at regular intervals since he left hospital care. A week after discharge he was very cheerful, described his past illness as like a dream, but was not in any sense disturbed by the recollection of it. He had gained a further 2 lbs. in weight. I saw him again in a fortnight (at this time the dose was reduced to 6 mgm. daily), and a month after discharge when his progress was well maintained. At the end of seven weeks he had put on $2\frac{1}{2}$ stones since the beginning of his illness, and was very fit indeed both mentally and physically, and commented himself on his own vastly improved powers of mental concentration and physical endurance. While previously rather pale, he now has a fresh complexion. After seven weeks the dose was reduced to 6 mgm. every other day. His weight and complete mental recovery were maintained when I saw him after fourteen weeks, when the dose was reduced further to 4 mgm. every alternate day.

An interesting feature in this case was the occurrence, after five months' treatment, of slight but definite œdema in both ankles and feet. This was most probably due to the water retention which occurs in some cases after treatment with testosterone. The patient had marked peripheral circulatory deficiencies, accounted for by premature cardiovascular degeneration and vasomotor instability. After five months' treatment he developed a red papular rash on the abdomen where he had rubbed in the ointment, but whether this was a specific effect (unlikely in that it developed after five months' of treatment), or whether contributed to by the effect of friction, one cannot say with certainty. It cleared up in a week or two.

**Case 3.** The patient, a woman aged 57, was admitted in a state of agitation and extreme depression. Sometimes she made monosyllabic statements, but mostly she was crying piteously and groaning loudly, expressing the delusion that she had killed her father. Before being put to bed she held herself bent double and had to be supported by two nurses when she tried to walk. Physically she was very ill. Her appearance was toxic and cachectic. No malignant condition was discovered. She was also investigated for pernicious anæmia with negative results. Her heart sounds were toneless, suggesting marked impairment of myocardial activity. One of the most striking features of this patient was her skin which was dry, yellow, excessively wrinkled, coarse and inelastic and resembling that of a very aged person. Though a woman of 5 ft. 10 ins. she weighed only 7 stone 9 lbs. on admission, which weight was reduced after a fortnight to 7 stone 7 lbs. After admission she had a period of restlessness and agitation, coupled with complete negativism to all necessary attention, and being very difficult about taking her food. She was abnormally affected by the war, alleging that Hitler had arrived already in Bailbrook and was in her room. She ran about shamelessly in the nude. She became very excitable, aggressive and abusive and used to attack members of the staff and throw water over them without provocation. She was incontinent and used to spit incessantly on the floor and her bed. She was very destructive of her own property and that of others and repeatedly destroyed bedclothes and nightdresses. She had bizarre delusions. For example, she avowed that her head had been bleeding all one afternoon and that Captain so-and-so had refused to let her be taken to hospital. Her resistiveness increased and she displayed definite catatonic gestures.

A month after admission she was put on Testoviron (Schering) ointment, 2·8 mgm. daily. The ointment was rubbed in according to the same technique as previously described. Her weight was then 7 stone 7 lbs. In two days she was taking her food better and was much quieter, but was still restless, agitated and depressed at night. Within the first week her delusions disappeared, she became co-operative and unresisting by day, and by the end of the first week she was no longer restless at night. A most remarkable feature was the improvement in her skin, which was very noticeable even at the end of the first week. It became more moist, clear, supple and less wrinkled.

At the end of a fortnight she could not be said to have any mental symptoms at all, except that she was diffident and rather abnormally unassertive. She kept herself productively occupied at knitting, etc., and went out walking. Her physical health had greatly improved. She looked far less toxic, but she weighed only 7 stone 6 lbs. and had, in fact, lost 3 lbs. since admission. After three weeks on the treatment the dose was increased to 5·6 mgm. testosterone daily. Within three weeks the change in her skin was miraculous. I do not think it would be any exaggeration to say that it looked forty years younger. One hesitated to employ such extravagant adjectives to her condition as a whole, but I think in view of the profound character of her symptoms just three weeks previously her mental improvement at this stage was remarkable. In her third week she put on 4 lbs., so that she weighed 7 stone 10 lbs. In the next week her weight had risen another 5 lbs., to 8 stone 1 lb.

She was discharged from hospital twenty-nine days after beginning treatment with Testoviron ointment. She had, in fact, recovered in a month from an acute psychotic condition accompanied by a general physical disturbance of such magnitude that I considered that maintainance of such agitation as she showed, taken in conjunction with her physical condition, would have had a fatal termination. I saw this patient again fourteen days after discharge. She had continued to have the same dose of ointment. She was extraordinarily well mentally, and much improved physically. She was alert, cheerful, and full of self-confidence about the future. Her weight had increased 1 stone 1 lb. in six weeks. The same dose was now given every other day and, when seen three weeks later, her remarkable mental improvement was maintained. She remained more or less the same weight as three weeks' previously, though I fancy she might well have gained further had she not been somewhat preoccupied with ideas of keeping slim.

I have investigated this patient's previous history very carefully. In the late autumn of 1918 she had, after influenza, a neurasthenic breakdown lasting six months and consisting chiefly of tremors and exhaustion. In 1929 she had a second attack, similarly psycho-neurotic, and lasting about three months. The patient is absolutely positive that the recent attack is infinitely more severe than these others she has experienced, and that her complete recovery has been attained in a vastly shorter time. It must be pointed out, too, that there is no indication that the two previous attacks were anything other than psycho-neurotic, though it is beyond all doubt that the third attack was psychotic, and of great intensity.

Making every allowance for spontaneous remissions in psychotic conditions, it is impossible that the improvement in these cases could be due to anything other than the hormone therapy. The second male patient was completely at a standstill. One of the most remarkable features of his case was the way his improvement kept pace with the rising dosage of Perandren. In the case of the female patient it can be argued that such acute symptoms as have been described are sometimes followed by a quick subsidence to normal. But for months before admission this woman had shown subacute psychotic symptoms. From her history prior to admission it is extremely unlikely that her condition could show such a remarkable improvement in so short a time. Her improvement was so dramatic that it was possible to notice it within a day or two of commencing treatment with Testoviron. While in her case the dosage of testosterone was not raised in so many successive steps as in the case of the male patient, the acceleration of her improvement on the increase of dosage was very noticeable.

None of these patients received any treatment, except sedatives when necessary for insomnia, etc. The response of these patients to inunction with testosterone shows that this hormone is absorbed very actively and in considerable dosage through the skin. In addition the progressive and peculiarly steady physical and mental improvement indicates a very even rate of absorption, not experienced in giving this hormone by injection.

